# The crossroads for patients: idealised nostalgia vs scientific evidence of past health

**DOI:** 10.1007/s11739-025-04156-2

**Published:** 2025-11-06

**Authors:** Francesco Maria Galassi, Elena Varotto

**Affiliations:** 1 Department of Anthropology, University of Lodz, Łódź, Poland; 2 School of Biomedicine, The University of Adelaide, Adelaide, SA Australia; 3 College of Humanities, Arts and Social Sciences, Flinders University, Adelaide, SA Australia

**Keywords:** Palaeopathology, History of medicine, Life expectancy, Communicable diseases, Vaccination, Preventive medicine

## Abstract

Patients sometimes recall a supposedly healthier past, imagined as a time when life was “natural” and free of disease. Such views, often amplified by anti-science movements, collapse when confronted with demographic and palaeopathological evidence. Pre-modern societies were marked by high infant and maternal mortality, recurrent epidemics, chronic malnutrition, and a life expectancy at birth rarely exceeding 35 years. Skeletal and mummified remains reveal tuberculosis, leprosy, syphilis, anaemia, malnutrition, trauma, and degenerative joint disease, underscoring the relentless burden of illness. By contrast, the remarkable rise in survival during the twentieth century resulted not from traditional diets or rustic lifestyles but from sanitation, vaccination, antibiotics, and preventive medicine. Life expectancy in the United States rose from 47 years in 1900 to nearly 79 years by the end of the century, with similar trajectories across Europe. Romanticising antiquity risks eroding confidence in biomedical progress, whereas palaeopathology underscores science as the true foundation of extended human longevity.

Patients often recall an allegedly healthier past, described nostalgically as a time when “everything was natural, organic, and free of disease.” Such recollections are not merely individual memories but rather cultural constructs, a form of collective yearning for an imagined simplicity. They are often amplified by anti-science ideological groups, which frame modern medicine as an artificial intrusion into a once “pure” human existence. Yet this narrative collapses under the weight of demographic, epidemiological, and palaeopathological evidence, which clearly and consistently documents that pre-modern societies were not havens of wellness but landscapes of relentless biological adversity.

Far from idyllic, life in pre-industrial communities was precarious and fragile. Infant and maternal mortality rates were extraordinarily high, with childbirth itself among the leading causes of death for women. Epidemics struck with alarming regularity, devastating entire settlements, while famine and chronic undernutrition ensured that survival was uncertain even in years without contagion. Malnutrition, endemic across social classes, compounded vulnerability to infectious agents. Life expectancy at birth seldom exceeded 35 years [1], meaning that for every individual who reached maturity, many more perished in infancy or early childhood. Those who did survive carried lifelong scars of hardship. Chronic degenerative joint diseases such as osteoarthritis, osteophytosis, and intervertebral disc herniations are visible in virtually all skeletal series from antiquity, attesting to the relentless biomechanical strain imposed by heavy physical labour and the absence of ergonomic relief [6, 7]. Daily subsistence—ploughing, hauling water, grinding grain, carrying fuel—placed enormous cumulative stress on the human body, making “natural life” less an idyll than an unending confrontation with wear, injury, and disease.

By contrast, the extraordinary leap in survival and wellbeing that took place in the twentieth century was not the result of “ancestral diets” or rustic lifestyles but of entirely modern achievements: sanitation, vaccination, antibiotics, and preventive medicine [2, 3]. These interventions collectively dismantled the ancien régime of high mortality. In the United States, for instance, life expectancy rose dramatically from 47 years in 1900 to nearly 79 years by the century's end [4]. Comparable trajectories unfolded across Europe, including Italy, where mass immunisation campaigns and the development of robust public health infrastructures reshaped mortality profiles and redefined the experience of aging [5]. What had once been perceived as inevitable—children lost in infancy, mothers dead in childbirth, adults broken by toil—became exceptional, largely prevented outcomes of an earlier epidemiological regime.

Palaeopathology, the study of disease in antiquity, provides perhaps the most eloquent and unassailable rebuttal to the myth of a disease-free past. Analyses of skeletal and mummified remains consistently reveal evidence of tuberculosis, leprosy, syphilis, metabolic disorders, nutritional deficiencies, traumatic injuries, and a wide spectrum of degenerative pathologies [6, 7]. The signs of morbidity are written into bone and tissue: *cribra orbitalia* and porotic hyperostosis, often reflecting chronic anaemia; enamel hypoplasia, betraying childhood stress episodes; periosteal reactions, recording infection and inflammation. These are not occasional findings but systematic, recurring patterns across populations, demonstrating that suffering and disease were woven inseparably into the fabric of daily existence. Palaeoepidemiology, by quantifying such lesions on a population scale, further confirms that disease burdens long predated industrialisation or modern environmental change [8].

Equally important is what pre-modern societies lacked: prevention as a public health concept did not exist. Information did not circulate with the immediacy of today’s digital age. There were no social networks to broadcast warnings, no newspapers to provide timely epidemiological updates. Communities often remained unaware of contagion until mortality was already widespread, and even then, the underlying causes were seldom understood. The absence of surviving reports in written sources must not be misconstrued as evidence that disease was absent. Absence of proof, palaeopathology reminds us, does not equate to proof of absence.

It is paradoxical that many individuals today—who often thrive into their seventh, eighth, or even ninth decade of life—attribute their longevity not to biomedical progress but to imagined virtues of the past. Such romanticising of antiquity does more than distort historical reality: it risks undermining public confidence in vaccines, preventive measures, and scientific innovation precisely at a moment when such trust is both fragile and indispensable. The persistence of this myth feeds into broader currents of medical scepticism and provides fertile ground for misinformation.

In this respect, palaeopathology does more than enrich medical history; it actively punctures illusions. It reminds us that the true “crossroads for patients” lies not between science and nostalgia, but between reality and myth. The difference between short, disease-plagued lives of the past and the extended survival of the present is not explained by lost traditions or forgotten diets but by the triumph of science. And it is science—not an idyllic but fictitious past—that has lifted human beings beyond the horizon of relentless morbidity. The task of science is to hold high the banner of truth; the responsibility of politics is to safeguard the conditions that allow science to remain above partisan distortions, free to illuminate the path forward (9).

## Data Availability

Not applicable.
